# Dangers of Celluloid

**Published:** 1890-03

**Authors:** 


### Dangers of Celluloid.

A curious accident from the combustion of celluloid, reported in the *Bulletin Medical*, June 2, 1889, may serve to call attention to the dangers connected with its use in the household. It seems that a girl, wearing a hair-comb of celluloid, was working near a very hot fire, when all at once her head was seen to be enveloped in flames. These were promptly extinguished, but not until the child was severely burned. The circumstances of the accident were inquired into by M. Leon Faucher, and made the basis of an interesting report to the Council of Hygiene. From this report we gather that celluloid is made from gun-cotton, by treating the latter with alcohol, then mixing with it some camphor, and subjecting the whole to considerable pressure. The temperature at which combustion takes place is relatively low—about four hundred degrees Fahrenheit. Light celluloid, that is, celluloid to which no coloring matters have been added—ignites at about fifty or sixty degrees higher. As regards the character of the combustion, it is said to be almost instantaneous, and to be accompanied with no light.

The accident in the case referred to must be regarded as unique, especially in the method of its production; for while it is quite possible that a person wearing an article of celluloid might approach dangerously near an exposed gas-jet, or stoop carelessly over a lamp, it can happen but rarely that, with such an article on, he or she would be near a fire hot enough to ignite it. Faucher, indeed, has been unable to find any record of a similar case.

Celluloid is now used for making so many things designed for domestic or toilet use, such as knife and fork handles, combs, backs of hair-brushes and hand-mirrors—that it can not be amiss to urge caution in bringing them near a fire, especially since several fatal accidents have been reported. A careless or ignorant house-maid—who is as likely as any one to have about her objects made of celluloid, if only collars and cuffs—might be the means of inflicting great damage upon herself or others. Of course this class of persons can not be reached through a medical journal, except very indirectly; but it would be well within the province of the family physician, in making his rounds and seeing articles of the kind described, to refer to the possible danger lurking in their use; and he could do this without either offending the intelligence of his patients or unduly exciting their fears.

